# First Reported Cases of SARS-CoV-2 Infection in Companion Animals — New York, March–April 2020

**DOI:** 10.15585/mmwr.mm6923e3

**Published:** 2020-06-12

**Authors:** Alexandra Newman, David Smith, Ria R. Ghai, Ryan M. Wallace, Mia Kim Torchetti, Christina Loiacono, Laura S. Murrell, Ann Carpenter, Scott Moroff, Jane A. Rooney, Casey Barton Behravesh

**Affiliations:** ^1^New York State Department of Public Health; ^2^New York State Department of Agriculture and Markets; ^3^COVID-19 One Health Working Group, CDC; ^4^National Center for Emerging and Zoonotic Infectious Diseases, CDC; ^5^National Veterinary Services Laboratories, Animal and Plant Health Inspection Service (APHIS), U.S. Department of Agriculture (USDA); ^6^Antech Diagnostics; ^7^One Health Coordination, APHIS, USDA.

On April 22, CDC and the U.S. Department of Agriculture (USDA) reported cases of two domestic cats with confirmed infection with SARS-CoV-2, the virus that causes coronavirus disease 2019 (COVID-19). These are the first reported companion animals (including pets and service animals) with SARS-CoV-2 infection in the United States, and among the first findings of SARS-CoV-2 symptomatic companion animals reported worldwide. These feline cases originated from separate households and were epidemiologically linked to suspected or confirmed human COVID-19 cases in their respective households. Notification of presumptive positive animal test results triggered a One Health* investigation by state and federal partners, who determined that no further transmission events to other animals or persons had occurred. Both cats fully recovered. Although there is currently no evidence that animals play a substantial role in spreading COVID-19, CDC advises persons with suspected or confirmed COVID-19 to restrict contact with animals during their illness and to monitor any animals with confirmed SARS-CoV-2 infection and separate them from other persons and animals at home (*1*).

SARS-CoV-2 is a zoonotic coronavirus that likely originated in bats (*2*). A small number of animals worldwide, including dogs, cats, zoo tigers and lions, and farmed mink, have been infected naturally with SARS-CoV-2, mostly through suspected human-to-animal transmission^†^ (*3*). In addition, experimental studies in ferrets, golden Syrian hamsters, Egyptian fruit bats, and cats show that these species can transmit infection to cohoused animals of the same species (*4*–*7*).

## SARS-CoV-2 Clinical Presentation in Domestic Cats

On March 24, in Nassau County, New York, a 4-year-old male domestic shorthair (cat A), developed respiratory illness characterized by sneezing, clear ocular discharge, and mild lethargy (Figure). On April 1, the cat was taken to a veterinary clinic; on physical examination the cat was found to be overweight, with a normal body temperature (101.4°F [38.6°C]). Nasal, oropharyngeal, and ocular swabs were collected by veterinary staff members and submitted to a private diagnostic laboratory (laboratory A) for a routine feline respiratory polymerase chain reaction (PCR) panel designed to detect *Mycoplasma felis, Bordetella bronchiseptica,* feline calicivirus, *Chlamydophila felis,* feline herpesvirus, and influenza A H1N1pdm. A broad-spectrum cephalosporin class antibiotic (cefovecin; 52 mg) was administered subcutaneously, and the cat was returned home, where it fully recovered by April 3. Results of the routine feline respiratory panel were negative for all pathogens and the specimen was tested using a SARS-CoV-2 reverse transcription PCR (RT-PCR) diagnostic assay as part of laboratory A’s passive COVID-19 pet surveillance program.

**FIGURE:**
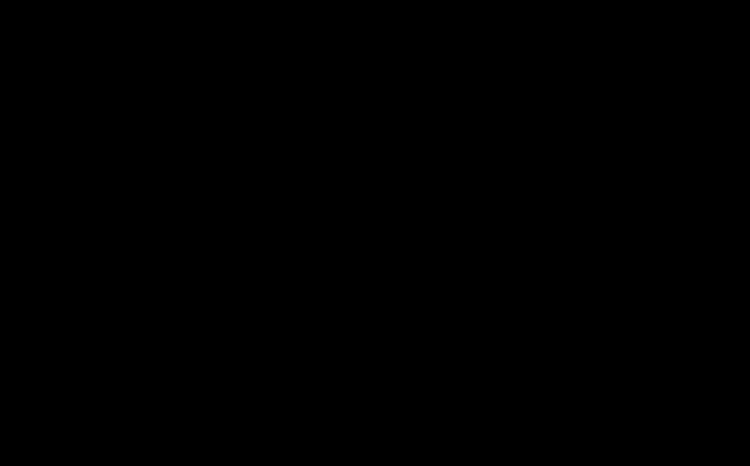
Timeline of events related to SARS-CoV-2 infections in two domestic cats (cats A and B) kept as pets in two different households — New York, March 15–April 22, 2020 **Abbreviations: **COVID-19 = coronavirus disease 2019; USDA NVSL = United States Department of Agriculture National Veterinary Services Laboratories.

On April 1, in Orange County, New York, a 5-year-old female Devon Rex (cat B), developed respiratory illness including sneezing, coughing, watery nasal and ocular discharge, loss of appetite, and lethargy. On April 6, the owner, an employee at a Connecticut veterinary clinic, collected conjunctival, nasal, deep oral, and fecal specimens from cat B in the home using sterile culturettes. These specimens also were sent to laboratory A and tested using the feline respiratory PCR panel. Cat B fully recovered by April 8 without treatment. At laboratory A, the feline respiratory PCR panel had a positive result for *Mycoplasma felis* and negative results for other common feline respiratory pathogens. The specimens from cat B also were tested by laboratory A for SARS-CoV-2.

On April 14, laboratory A reported a positive SARS-CoV-2 RT-PCR result for cat A to the USDA National Veterinary Services Laboratories (NVSL), veterinary clinic, and New York state veterinarian, who immediately notified the New York State Department of Health (NYSDH). The same day, laboratory A notified NVSL and Connecticut state animal health officials of the positive SARS-CoV-2 RT-PCR result for cat B. After determining that cat B resided in New York, the New York state veterinarian was informed, and the NYSDH was immediately notified. RNA from the positive respiratory specimens from both cat A and cat B were forwarded from laboratory A to NVSL for confirmatory testing.

## Public Health Response

On April 14, following notification of presumptive positive SARS-CoV-2 test results for cats A and B, state and federal partners conducted a joint epidemiologic investigation. Household members and veterinarians who had treated the infected cats were questioned regarding the cats’ living arrangements, health condition, potential sources of infection, and risks posed by these animals to other animals inside and outside the home, and to humans.

Cat A lived in an apartment with five persons, including three who had shown signs of mild respiratory illness including fever, cough, and sweating; none of the five were tested for SARS-CoV-2 infection. The first person’s illness began around March 15, 9 days before cat A became ill, and lasted <48 hours. Residents of the household’s apartment complex also experienced multiple cases of human COVID-19 around the same time. A second cat in the household, a 3-year-old female domestic shorthair, remained healthy and was not tested for SARS-CoV-2. Both cats were typically kept indoors but did occasionally venture outside.

Cat B lived in a single-family home with one person, who developed fever, productive cough, chills, muscle aches, abdominal pain, headache, diarrhea, sore throat, and fatigue on March 24, 8 days before cat B became ill. Specimens collected from this person on March 26 for viral testing were positive for SARS-CoV-2. By March 27, the illness had resolved. A second cat in the household, a 7-year-old Devon Rex, remained healthy and was not tested for SARS-CoV-2. Both cats were kept exclusively indoors.

On April 17, state and local One Health partners collected additional specimens from cats A and B for confirmatory diagnosis of SARS-CoV-2 at NVSL (Table). Real-time RT-PCR, using a modified CDC N-target assay and sequencing (*8*), determined that results for both cat A and B were positive at the first specimen collections (April 1 and 6, respectively), and the nasal swab from cat A was weakly positive from the subsequent collection (April 17). Both cats had SARS-CoV-2–specific virus neutralizing antibodies, but virus isolation in cell culture from subsequent specimen collection was unsuccessful for both cats, likely due to virus clearance. Cat A and B recovered from illness 11 days and 6 days before initiation of the epidemiologic investigation; therefore, no additional monitoring or infection prevention measures were recommended.

**TABLE:** Results of SARS-CoV-2 real-time RT-PCR, partial next-generation sequencing, SARS-CoV-2 virus neutralization, and virus isolation in two domestic cats kept as pets (cat A and cat B) by specimen type and date collected — U.S. Department of Agriculture National Veterinary Services Laboratories, United States, April 2020

Case	Date collected	Specimen type	N1* target result (Average Ct)^†^	N2* target result (Average Ct)^†^	Spike gene sequencing	Virus neutralization	Virus isolation
Cat A	April 1	Laboratory A-extracted RNA	Positive (22.3)	Positive (24.4)	Positive	N/A	N/A
	April 17	Nasal swab	Positive (35.9)	Positive (37.3)	Positive	N/A	Negative
	April 17	Rectal swab	Negative	Negative	N/A	N/A	Negative
	April 17	Serum	N/A	N/A	N/A	Positive	N/A
Cat B	April 6	Laboratory A-extracted RNA	Positive (27.1)	Positive (26.2)	Positive	N/A	N/A
	April 17	Nasal swab	Negative	Negative	N/A	N/A	Negative
	April 17	Rectal swab	Negative	Negative	N/A	N/A	Negative
	April 17	Serum	N/A	N/A	N/A	Positive	N/A

Abbreviations: Ct = cycle threshold; N1 = virus nucleocapsid gene 1; N2 = virus nucleocapsid gene 2; N/A = not applicable; RT-PCR = reverse transcription–polymerase chain reaction.

* N1 and N2 targets = primer-probes for CDC’s real-time RT-PCR assay that targets virus nucleocapsid (N) gene for specific detection of SARS-CoV-2.

**^†^** Ct = the number of cycles required for the fluorescent signal to cross the threshold, where lower values indicate more starting nucleic acid.

## Discussion

An estimated 76 million pet cats live in the United States, and approximately 70% of U.S. households own at least one pet (*9*). Close interactions between humans and pets create opportunities for zoonotic disease transmission. In both cases presented in this report, the cats with positive test results for SARS-CoV-2 had close epidemiologic links to owners with suspected or confirmed COVID-19. In addition, human symptom onset preceded that in cat A by 9 days and in cat B by 8 days. No identified onward human or animal infections were attributed to these animals. This evidence supports findings to date that animals do not play a substantial role in spreading SARS-CoV-2, although human-to-animal transmission can occur in some situations. Companion animals that test positive for SARS-CoV-2 should be monitored and separated from persons and other animals until they recover.

Both animals in this report were initially tested by laboratory A as part of a passive COVID-19 pet surveillance program that operated independently from state and federal health agencies. This method of surveillance was unable to routinely obtain epidemiologic information regarding SARS-CoV-2 exposures before testing. CDC and USDA have identified four situational testing categories^§^ (*10*); one of the four categories recommends testing symptomatic animals with close contact to a person with suspected or confirmed COVID-19. Epidemiologic investigation conducted after positive SARS-CoV-2 test results were reported found that both cat A and cat B fit this situational category.

Currently, CDC and USDA recommend that epidemiologic information be collected before companion animal SARS-CoV-2 testing, and that the decision to test animals be coordinated with state public health veterinarians and state animal health officials using a One Health approach, to ensure that animal and public health responses occur in a timely and effective manner. Laboratory A’s passive surveillance program operated for a limited period to better understand the impact of SARS-CoV-2 on animals at risk for infection and did not divert resources necessary to conduct human SARS-CoV-2 testing, consistent with CDC and USDA guidance.

Establishment of the U.S. One Health Federal Interagency COVID-19 Coordination Group (OHFICCG) in February 2020, and routine communication between state and federal One Health partners have been instrumental in ensuring a coordinated government response to the One Health aspects of COVID-19. This One Health coordination platform allows for collaboration and rapid information-sharing across sectors while also facilitating alignment of research, priorities, and messaging regarding the human, animal, and environmental aspects of COVID-19. Laboratory A, state partners, and members of OHFICCG coordinated information sharing during this investigation. Information from this investigation informed OHFICCG guidance development for managing SARS-CoV-2–infected animals, including guidance for when animals with positive test results should resume normal activities. This investigation provides further support for the utility of a One Health approach to addressing zoonotic diseases such as COVID-19 to safeguard the health, welfare, and safety of humans, animals, and their shared environment.

**Summary**

**What is already known about this topic?**

A small number of companion animals worldwide have been naturally infected with SARS-CoV-2, the virus that causes COVID-19.

**What is added by this report?**

Two domestic cats with respiratory illnesses lasting 8 and 10 days are the first reported companion animals with SARS-CoV-2 infection in the United States. Both cats were owned by persons with suspected or confirmed COVID-19, and both cats fully recovered.

**What are the implications for public health practice?**

Human-to-animal transmission of SARS-CoV-2 can occasionally occur. Animals are not known to play a substantial role in spreading COVID-19, but persons with COVID-19 should avoid contact with animals. Companion animals that test positive for SARS-CoV-2 should be monitored and separated from persons and other animals until they recover.

## Footnotes

* One Health is a collaborative, multisectoral, and transdisciplinary approach, working at the local, regional, national, and global levels, with the goal of achieving optimal health outcomes recognizing the interconnection between humans, animals, plants, and their shared environment.

† https://www.oie.int/scientific-expertise/specific-information-and-recommendations/questions-and-answers-on-2019novel-coronavirus/.

§ Testing is indicated for four situational categories: 1) Animals with clinical signs of illness consistent with SARS-CoV-2 infection and an epidemiologic link to a person with suspected or confirmed COVID-19; 2) Animals with clinical signs of illness consistent with SARS-CoV-2 infection and an epidemiologic link to an environment that is at high risk for SARS-CoV-2 contamination; 3) Threatened, endangered, or otherwise imperiled or rare animals in a rehabilitation or zoologic facility with possible exposure to SARS-CoV-2 through an infected person or animal; 4) Animals in a mass care or group setting where a cluster of animals shows clinical signs of illness consistent with SARS-CoV-2.
